# Organizational impact of evidence-informed decision making training initiatives: a case study comparison of two approaches

**DOI:** 10.1186/1748-5908-9-53

**Published:** 2014-05-02

**Authors:** François Champagne, Louise Lemieux-Charles, Marie-France Duranceau, Gail MacKean, Trish Reay

**Affiliations:** 1 Departement d’administration de la santé, Université de Montréal, Montreal, Quebec, Canada; 2 Institute of Health Policy, Management and Evaluation, University of Toronto, 155 College St., 4th floor, Toronto, ON M5T 3 M6, Canada; 3 Department of Community Health Sciences, University of Calgary, Calgary, Alberta, Canada; 4 Alberta School of Business, University of Alberta, Edmonton, Alberta, Canada

**Keywords:** Theory-driven evaluation, Organizational learning, Knowledge creation, Evidence-informed decision making, Healthcare organizations

## Abstract

**Background:**

The impact of efforts by healthcare organizations to enhance the use of evidence to improve organizational processes through training programs has seldom been assessed. We therefore endeavored to assess whether and how the training of mid- and senior-level healthcare managers could lead to organizational change.

**Methods:**

We conducted a theory-driven evaluation of the organizational impact of healthcare leaders’ participation in two training programs using a logic model based on Nonaka’s theory of knowledge conversion. We analyzed six case studies nested within the two programs using three embedded units of analysis (individual, group and organization). Interviews were conducted during intensive one-week data collection site visits. A total of 84 people were interviewed.

**Results:**

We found that the impact of training could primarily be felt in trainees’ immediate work environments. The conversion of attitudes was found to be easier to achieve than the conversion of skills. Our results show that, although socialization and externalization were common in all cases, a lack of combination impeded the conversion of skills. We also identified several individual, organizational and program design factors that facilitated and/or impeded the dissemination of the attitudes and skills gained by trainees to other organizational members.

**Conclusions:**

Our theory-driven evaluation showed that factors before, during and after training can influence the extent of skills and knowledge transfer. Our evaluation went further than previous research by revealing the influence—both positive and negative—of specific organizational factors on extending the impact of training programs.

## Background

‘Despite the purported focus of theory-based evaluation on investigating the causal mechanisms by which a program achieves its effects, surprisingly few actually do this’ [1].

Over the past 20 years, organizational learning and knowledge have come to be widely considered as important determinants of organizational change and performance [2]. On this account, learning and knowledge are taken to be sources of competitive advantage, and many experts consider the ability to acquire, create and use knowledge to be the most important source of an organization’s sustainability [3]. In healthcare organizations, the challenge is especially acute and is linked to both care quality and service efficiency.

Many theorists have emphasized the need for increased attention to and mobilization of evidence-informed decision making (EIDM) to support management practices in healthcare organizations [4,5]. The underlying premise is that the use of scientific evidence should lead to higher quality decisions, to the implementation of higher quality actions and, consequently, to better outcomes. Based on this premise, healthcare organizations and health system leaders have made significant efforts to encourage the use of evidence in decision making, believing it will lead to more effective organizational management; as a result, many different strategies have been formulated to facilitate healthcare managers’ use of EIDM. The impact of those efforts on actual practices within organizations is, however, far from clear.

In Canada, two national organizations—the Canadian Health Services Research Foundation (CHSRF) and SEARCH Canada—developed health service executive training programs focused on helping managers develop the skills needed to acquire, appraise, adapt and apply research results. On 5 November 2012 (after we had concluded our research), the Canadian for Health Services Research Foundation changed its name to the Canadian Foundation for Healthcare Improvement (CFHI). SEARCH Canada ceased operation on 30 September 2009, after we had begun our research. We decided, however, to pursue our SEARCH-related inquiries because the end of the program did not undermine the relevance of evaluating its organizational impact up to that terminus.

The CHSRF’s Executive Training for Research Application (EXTRA) program, which is still ongoing, aims ‘to facilitate the spread of evidence-informed health system management throughout senior levels until a critical mass is achieved in the system’ [6]. SEARCH Canada had a similar objective for its SEARCH (Swift, Efficient Application of Research in Community Health) Classic program: to help healthcare organizations apply new knowledge to make sound decisions by building strong collaborative relationships, sharing information and developing people [7]. While the impact of those programs on individuals has been repeatedly evaluated, their organizational impact remains unclear (in fact, there is little empirical evidence in the literature on the organizational impact of such training programs in general).

We therefore conducted a theory-driven evaluation focused on understanding the organizational impact of having healthcare leaders take part in either EXTRA or SEARCH Classic. In our work, we interpreted those programs as novel knowledge conversion strategies that emphasize the reinforcement of organizational culture and knowledge use processes through the training of decision makers.

In this article, we first describe the context of the two programs and the methods we used to evaluate their organizational impact. We then present our findings of their impact and assess the processes through which it occurred. Next, we discuss the contextual conditions that facilitated or impeded the use of new knowledge, and our final section summarizes the main points and principal lessons for organizational capacity building.

## Training programs

### EXTRA

Many resources have been allocated to enhance the use of EIDM in healthcare organizations. In 2004, the CHSRF developed the EXTRA program for senior managers in Canada. While it has evolved since then, it had two objectives at the time of our research in 2008: to increase the skills of health service professionals selected as EXTRA fellows in using research to manage Canada’s healthcare system more effectively; and to encourage health service professionals selected as EXTRA fellows to collaborate in the management of healthcare delivery.

Designed to be a long-term initiative, EXTRA was expected to produce a significant number of motivated health service professionals who would be equipped with the skills required to use research in order to improve the quality and effectiveness of Canada’s healthcare system. EXTRA’s underlying—and still current—assumption is that the actions of and interactions among a substantial number of mid- and senior-level managers who have the skills, knowledge and desire to build organizational capacity for using evidence-informed knowledge should lead to a more systematic use of evidence in organizational decision making. This assumption likewise maintains that decision makers should also act as important agents of change within their organizations.

The EXTRA program had, at the time of our research, five core components:

1. Four away-from-home residency sessions.

2. One or more intervention projects at a fellow’s home organization, proposed when he/she applied to the program (intervention projects were presented to expert panels and organizations’ chief executive officers (CEOs) during the final session).

3. Educational activities between residency sessions.

4. Network-building opportunities among faculty and other fellows during the program (mentoring by individual faculty and mentoring teams was provided on site and in the periods between the residency session).

5. Post-program support and activities aimed at building an EIDM community of practice.

Self-directed learning was facilitated through the EXTRA Desktop. This was a customized internet-based learning platform that provided participants with an electronic classroom; a virtual library of online course software, databases, search engines, journals, and other resources, as well as a variety of Internet technologies; and a virtual environment for collaboration and dialogue with other participants, faculty and mentors. A post-program community of practice for fellows and organizations enabled EXTRA alumni to continue their professional development and to build networks of pan-Canadian decision makers and healthcare organizations with whom and through which to share knowledge and experiences in health services management and delivery.

### SEARCH classic

Until its termination in 2009, SEARCH Canada helped healthcare organization leaders apply new knowledge in an effort to make sound decisions. It did so by building strong collaborative relationships, sharing information and developing people. SEARCH Canada was committed to four main goals and practices: enhancing the growth of practicing professionals and applied researchers; collaborating with academic, service and government partners across the health system; working with both organizations and individuals; and working in ways that integrated capacity into the core business and activities of healthcare organizations.

SEARCH Classic was an intense, two-year experience that combined learning opportunities through face-to-face modules, inter-module work and the application of knowledge to practice-based projects. The three pillars of the SEARCH curriculum were choosing evidence, creating evidence and using evidence.

SEARCH Classic participants—called SEARCHers—came from across the Province of Alberta, and they had access to extensive knowledge management resources and tools. SEARCHers also relied on the support of a vibrant Alberta-wide network of talented individuals who championed the cause of applied research (conducted on a regional basis) and its application in healthcare organizations, and who remained connected to the program participants and continued to collaborate with them following the conclusion of their formal involvement with the program.

### EXTRA and SEARCH classic: similarities and differences

Despite their local differences, the overarching aims of EXTRA and SEARCH Classic were the same: to enhance organizational capacity to use EIDM. Their main operating hypotheses were also similar: in the context of strong organizational commitment, individual training should lead to organizational use of evidence. Table 1 presents the main features of the EXTRA and SEARCH Classic programs, focusing on their similarities and differences.

**Table 1:** Similarities and differences between EXTRA and SEARCH Classic

	**EXTRA**	**SEARCH Classic**
Number of trainees	24-28 fellows	27 SEARCHers (average)
Program duration	2 years	2 years
Number of years in operation	2004-present	1996-2009
Target clientele	Senior-level managers	Mid-level managers
Program foci	Skills in sound management and leadership, and in conducting and using research (more emphasis on management and leadership)	Skills in conducting and using research, and in sound management and leadership (more emphasis on research skills)
Intervention project	Linked to organizational strategy; conducted in and with organizations	Local projects: often a literature review and linked to an organizational priority; provincial projects: applied or linked to a provincial priority
Links with mentors	During fellowship	During and after fellowship
Scale	National	Provincial

### Theoretical framework

Both EXTRA and SEARCH Classic were designed to influence participants’ skills and knowledge. However, the programs also were intended to have a wider impact, and were founded on the assumption that the diffusion of knowledge would occur within trainees’ organizations. In order to gauge the extent to which the latter came to fruition, we sought to answer three questions:

1. What was the nature and extent of the impact on the organizations of having a number of mid- and senior-level managers trained through EXTRA or SEARCH Classic?

2. What were the organizational processes through which the programs’ impact occurred?

3. What were the contextual conditions that facilitated or impeded the programs’ impact?

**Figure 1:**
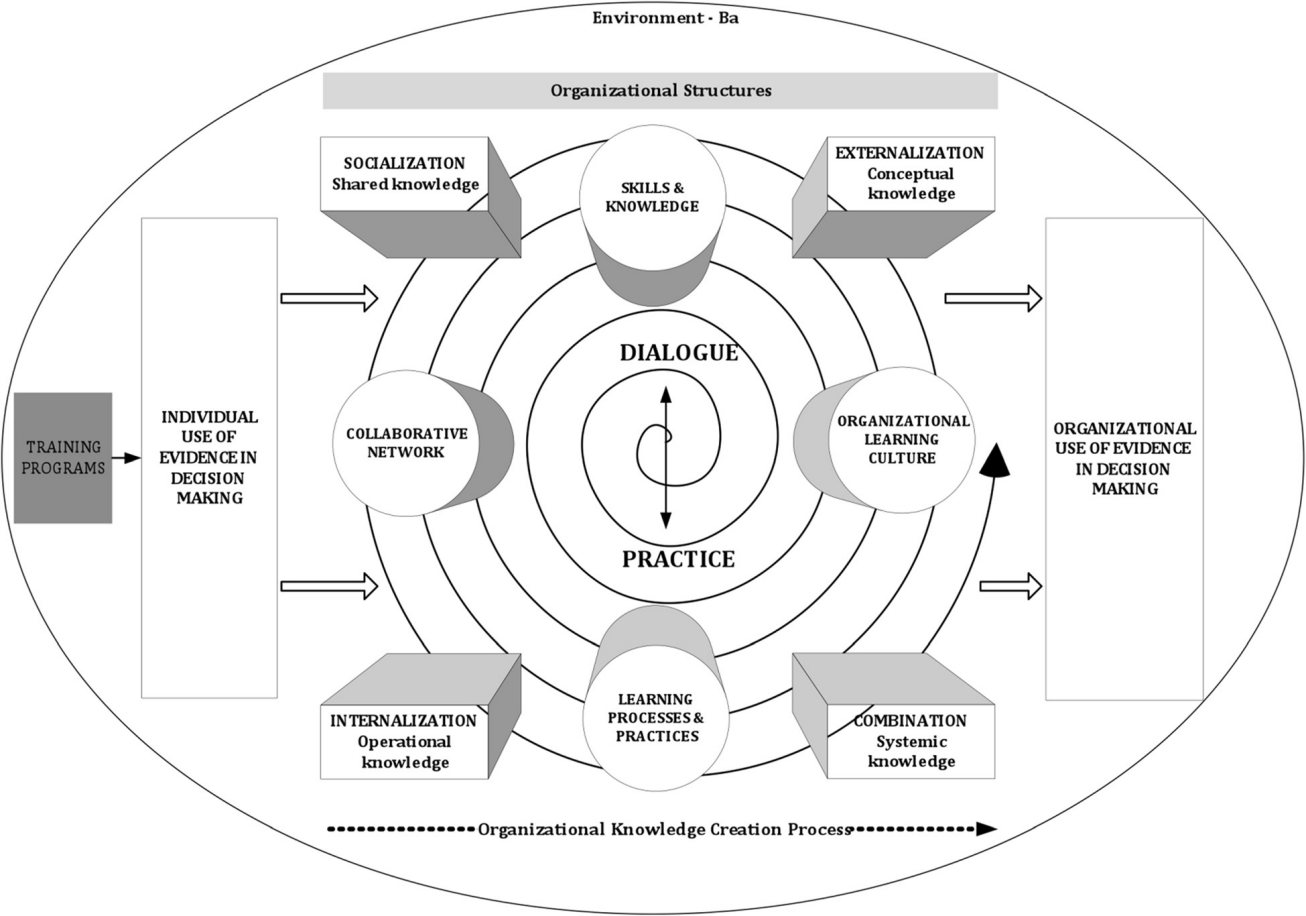
**Knowledge creation logic model** ***.***

To guide our work, we developed an integrated logic model (Figure 1). We based this model on several sources: Nonaka’s Dynamic Theory of Organizational Knowledge Creation [8-11]; Patton’s work on evaluation process use [12,13]; Cousins *et al.’s* Framework of Evaluative Inquiry as an Organizational Learning System [14]; and the Framework for the Analysis and Optimization of the Use of Scientific Evidence and Knowledge in Decision Making, from Champagne and Lemieux-Charles’ collection of essays examining EIDM in clinical, organizational and policy contexts [15]. We also incorporated into our model organizational-learning capacity (*i.e.*, an organization as a learning system) and organizational consequences (*i.e.*, shared mental representations); and we linked individual learning to organizational capacity building and learning.

According to our logic model, program participants who emerge with improved skills and knowledge, as well as with reinforced attitudes, intentions, and commitment, will use their tacit and explicit knowledge when interacting with other people within their organizations. We hypothesized, therefore, that the impact of the EXTRA and SEARCH Classic programs would occur through a dynamic process of knowledge creation, that would, in turn, strengthen the learning capacity of and processes in participants’ organizations.

Researchers have found that organization-level impact occurs through two dimensions of knowledge: tacit and explicit [8-11]. Rooted in action, experience and involvement in a specific context, the tacit dimension of knowledge refers to an individual's beliefs and viewpoints, as well as to his/her concrete context-specific skills. The explicit dimension of knowledge is articulated, codified and communicated in symbolic form and/or natural language.

Nonaka [8] and Nonaka and Toyama [16] regard organizational learning as a dynamic process of knowledge creation based on four modes of context-specific knowledge conversion: socialization (tacit to tacit), externalization (tacit to explicit), combination (explicit to explicit), and internalization (explicit to tacit):

Socialization is the process of converting new tacit knowledge through shared experiences and observations. New tacit knowledge is acquired when people spend time together (*e.g.*, by living in the same environment). It is acquired through discussions, interactions and observations. Exchange can be formal or informal.

Externalization is the process of transforming tacit knowledge into explicit knowledge, which occurs through the use of formal communication tools. This process—the articulation of knowledge—is largely about developing a common understanding of a problem, solution or situation.

Combination is the reconfiguration or construction of new knowledge into a more complex form. Explicit knowledge comes from inside or outside an organization and requires the involvement and participation of other people.

Internalization happens when explicit knowledge created and shared throughout an organization is then converted by individuals into tacit knowledge. When people internalize new knowledge, it becomes part of their tacit knowledge.

According to Nonaka [8] and Nonaka and Toyama [16], individuals initially create knowledge, which then becomes organizational through the process of knowledge conversion. This theory seemed appropriate to guide our evaluation because both EXTRA and SEARCH Classic implicitly assumed that the knowledge gained by participants would spread to other members of their organizations.

This theory of knowledge conversion posits that knowledge spreads out from an individual to his/her organization through spiraling interactions (which happen in specific organizational contexts) between tacit and explicit knowledge. The theory also takes into consideration the creation of knowledge through the dynamic phenomenon of the ba. Nonaka *et al.*[17] define this concept as the shared context—physical, mental or virtual—in which dialogues and practices take place in order to implement an organization’s vision and objectives. On this account, creating new knowledge requires shared emotion, mental models, experiences, strategy and vision.

### Variables

Our conceptual model posits that a number of organizational and environmental factors facilitate or impede the knowledge conversion process (see Figure 1). During our research, we therefore looked for the influence of those variables on knowledge conversion processes as well as on the organizational use of knowledge. Drawing on the literature and on the basis of our previous work [15], we defined the following 12 variables (eight organizational structures and 4 organizational learning characteristics):

## Organizational structures

### Organizational skills and knowledge stock

This variable concerns the level of accumulated knowledge in an organization. We drew our definition from the work of Polanyi [18] and, more recently, Nonaka [8] and Nonaka *et al.*[17]. Of the tacit dimension of knowledge, Alavi and Leidner follow Nonaka in arguing, ‘rooted in action, experience, and involvement in a specific context, the tacit dimension is comprised of both cognitive and technical elements. The cognitive element refers to an individual’s mental models, consisting of mental maps, beliefs, paradigms, and viewpoints. The technical component consists of concrete know-how, crafts, and skills that apply to a specific context’ [19].

### Organic structure

This variable is based on work on organizational structure conducted by Burns and Stalker [20] according to whom an organic structure is a facilitator for innovation (we consider EIDM to be such an innovation). For Burns and Stalker, organic structures have the following characteristics: low level of job formalization (*e.g.*, few rules and task descriptions), fluid and flexible network functioning, low level of hierarchy, low standardization of work processes, and decentralized decision making.

### Organizational communication

This variable is the degree to which information is transmitted among members of an organization [21].

### Innovation and learning-based reward system

This variable concerns the extent to which rewards are given based on demonstrated capacities to learn and innovate [22].

### Professional development activities

These are all activities of training and continuing education put in place by an organization for its employees.

### Knowledge system

This variable refers to all the systems implemented to promote and facilitate knowledge use in an organization [23,24]. These systems include information systems (*e.g.*, data collection, storage and transmission systems, access to scientific literature and reviews and knowledge broker positions (*e.g.*, librarian, knowledge consultant).

### Leadership

It is an essential function to prepare and mobilize organizational participants for change and to create a balance between exploitation of current capabilities and exploration and development of new capabilities. Leadership must be distributed broadly if organizations are to increase their capacity for learning and change and therefore to flourish in a complex and changing environment [25]. In our project, leadership is the ability to motivate others toward the use of EIDM.

### Strategy

Strategy is understood as a pattern in organizational decisions or actions [26]. In our research, we regarded a deliberate strategy for using EIDM to be a favorable condition.

## Organizational learning characteristics

### Skills and knowledge

This variable refers to individuals’ capacity to acquire, assess, apply and adapt evidence. Those four steps involved in the use of evidence are derived from the CFHI’s tool designed to help organizations create, share and use knowledge [27].

### Organizational learning culture

This variable refers to the extent to which individual and organizational learning is valued, integrated and rewarded in an organization. An organization with a strong learning culture will ensure that individual learning can be converted to organizational learning by providing a receptive milieu for individual learning and by putting in place appropriate mechanisms to enable, support, and reward the use of what is learned [28].

### Learning processes and practices

This variable is closely linked to organizational learning culture. The learning process is a cycle of action and reflection—namely, doing and thinking, performing and conversing [28].

### Collaborative network

This refers to the extent to which organizational participants’ work involves networking and collaboration both within and outside their organization.

## Methods

### Study design

We analyzed six case studies using three embedded units of analysis: individual, group and organization. Our analysis relied on a triple comparative design [29], whereby the relationships hypothesized in our logic model were first analyzed synchronically (at one point in time, measured as the general situation in the organizations); second, diachronically (longitudinally through tracer situations composed of the participants’ intervention projects as well as additional instances of decisions recently made in their organizations); and third, transversally (in parallel) across cases (participants, projects, decisions, and organizations).

Although our study involved six cases nested inside two programs, it was not designed to be a formal comparative analysis as understood in fields such as anthropology and political science. Rather, we used multiple case studies primarily to compare findings from each case [30], a method that enabled us to examine the mechanisms through which EXTRA and SEARCH Classic contributed to changes within participants’ six organizations.

### Case selection

We selected six cases in order to obtain a diverse mix of provinces and health systems (Alberta, Saskatchewan, Quebec and Nova Scotia); organizational type, size and complexity; urban and rural locations; and extent of participation in the two programs. The selection reflected different geographic and healthcare configurations across Canada. All provinces have some degree of regionalization of health services, although Alberta reverted back to a single authority in 2009. The EXTRA sites were all academic health centers, and two of the SEARCH Classic sites combined rural and urban locations. One SEARCH Classic site was entirely rural. Table 2 outlines the number of individuals within each case selected who had participated (up to 2008) in the EXTRA and SEARCH Classic programs.

**Table 2:** EXTRA and SEARCH Classic participants by province and setting

**Province**	**Number of program participants until 2008**		**Setting**
	**EXTRA**	**SEARCH**	
Alberta	3	18	Part-urban/part-rural
	0	23	Part-urban/part-rural
	0	8	Rural
Saskatchewan	3	1	Urban
Quebec	5	0	Urban
Nova Scotia	7	0	Urban

### Organizational contexts and EXTRA fellows/SEARCHers’ projects

According to our model, we expected EXTRA fellows’ and SEARCHers’ individual characteristics, organizational contexts and environments to influence the knowledge conversion process. The SEARCHers and EXTRA fellows had different educational backgrounds. and generally held positions as senior-level clinical and administrative leaders; the SEARCHers, however, were more likely to occupy clinical leader positions. Examples of individual projects included programs related to increasing healthcare efficiency and quality; for example, developing quality-of-life indicators, patient safety programs, stroke rehabilitation guidelines and approaches to increasing the efficiency and effectiveness of patient flow. The EXTRA and SEARCH Classic projects were different in scope: SEARCHers’ individual projects focused on literature reviews, whereas the EXTRA fellows’ projects were applied interventions. There were also differences in the degree to which the various projects were aligned with organizational strategies.

In all six of our cases, senior managers showed a strong commitment to the development of research capacity and utilization in their organizations. In one rural site, participation in SEARCH Classic was part of a strategic plan for capacity building across all units of the organization. In one urban specialist academic center, the organization’s official values included a clear commitment to EIDM as a means of bringing about innovation.

### Data collection

For each case, we collected data by interviewing EXTRA fellows and/or SEARCHers (individually, when numbers allowed; in groups of two or three, when numbers were large); supervisors (individually); colleagues, as selected by the supervisors and/or the EXTRA fellows/SEARCHers (either individually or in groups of two or three); and vice presidents and CEOs.

Interviews were conducted during intensive, one-week data collection site visits by the research coordinator; in four cases, a co-investigator accompanied the research coordinator. A total of 84 people were interviewed. Table 3 outlines the number of individuals interviewed by case as well as their organizational positions.

**Table 3:** Number of individuals interviewed by position and case

**Participants**	**Case 1**	**Case 2**	**Case 3**	**Case 4**	**Case 5**	**Case 6**	**Total**
EXTRA fellows	1	0	1	4	4	6	16
SEARCHers	6	11	6	0	0	0	23
Colleagues	5	6	1	1	7	7	27
Supervisors	1	1	5	0	4	2	13
Vice-presidents and CEOs	1	1	1	1	1	1	6
Total	14	19	14	6	16	16	

The interviews addressed the following areas: the first section focused on the individual and his/her experience with either EXTRA and/or SEARCH Classic; perceptions of individual and organizational use of EIDM; and perceptions of organizational support in the use of EIDM. The second section focused on the interviewees’ intervention projects. We used the intervention projects as tracers in order to analyze the knowledge conversion process. Data for all projects discussed were analyzed and further analysis was carried out on four projects where more in-depth information had been collected. This allowed us to analyze in more depth the conversion process. Three of the projects represented examples of a successful conversion project while the fourth one had been less successful.

We also collected data from available organizational documents (provided by the organizations or found through the organizations’ web sites), including strategic plans and intervention project reports. We searched these documents using the following key words: EXTRA or SEARCH, evidence, evidence-informed decision making, scientific data, knowledge, and decision making. This documentary analysis was used to determine whether formal mechanisms had been put in place to enhance the use of evidence in decision making.

### Analysis

The interviews were transcribed in their entirety. We first analyzed them using an open coding system with QDA Miner v3.0.3. The coding strategy used emergent and predetermined categories. Predetermined categories included socialization; externalization; internalization and combination; collaborative network; skills and knowledge; organizational learning culture and learning processes and practices; leadership innovation and learning based reward system; and knowledge system. Emergent categories, such as organic structure, strategy and organizational communication, were added to the original coding.

To establish reliability and add rigor to the process, the categories were discussed with the investigator group before starting the coding. The two principal investigators and the coordinator then independently coded an initial subset of transcribed interviews to assess coding consistency. Through discussion following the coding, disagreements were addressed and emergent categories identified. Interview notes and organizational documents were analyzed using the same codes.

The study was conducted between June 2009 and January 2010, and was approved by the Université de Montréal ethics committee (CERFM #342) and all six ethics committees of the healthcare organizations involved in the research. Participation in the research was voluntary and all participants signed a consent form.

## Results

### Use of EIDM as reported by EXTRA fellows and SEARCHers

Our main hypothesis was that the action and interaction of EXTRA fellows and SEARCHers would result in a dynamic knowledge creation process capable of reinforcing an organization’s learning capacity that would, thereby, lead to beneficial organizational outcomes. We first asked EXTRA fellows and SEARCHers about their understanding and use of evidence in decision making, aligning our questions with the four steps involved in the use of EIDM: acquiring, assessing, adapting and applying evidence to decision making. These questions uncovered distinct variations among sites and individuals. We defined ‘use’ of EIDM according to the CFHI’s self-assessment tool [27]: acquire: where to look for and access research; *assess:* the quality and relevance of research; adapt: summarizing and relating research to context; apply: how research recommendations inform decision making.

The interviews we conducted reaffirmed what is known from previous studies (*e.g.*, [31]): most participants believed they had good skills in acquiring and assessing evidence. Similarly, almost all the EXTRA fellows and SEARCHers across all the sites felt confident about their skills in acquiring, adapting and applying evidence. The interviewees perceived adaptation and application as the two easiest steps, and as the ones that were the most integral components of their managerial functions.

Across all sites, EXTRA fellows and SEARCHers reported barriers confronting their use of evidence. In three sites, these barriers were important enough to jeopardize the use of EIDM. The barriers participants identified related to organizational structure; more precisely, there were obstacles to accessing scientific literature databases (this was a particularly major challenge in the SEARCH Classic sites). In addition, access to human support (*e.g.*, librarians, experts) to facilitate the use of EIDM varied across the sites. Other barriers identified included data availability and data quality, as well as the EIDM process itself (*e.g.*, the time required to make a decision and the complexity of the process in non-clinical fields).

### Use of EIDM as reported by others in the organizations

According to our logic model, the knowledge conversion process should lead to a change in the use of EIDM at the organizational level. We therefore endeavored to assess changes in the use of evidence at that level by interviewing trainees’ colleagues. According to the people we interviewed, the use of EIDM varied considerably within their organizations, and we found evidence at all six sites that EIDM was, in fact, less extensive than our subjects cared to admit; for example, one interviewee remarked,

‘I think all professionals want to be able to say, ‘I use evidence when I’m practicing or making a decision.’ When the fellows start taking the fellowship, they come back and they say, ‘We’re not using the evidence we think we are.’ But, really, the scope of evidence is much greater than our own little personal view of what evidence is.’

When we compared responses from SEARCH site and EXTRA site non-program interviewees, the former group reported a more limited use of EIDM. In both sets of sites, however, our analysis indicated that non-fellows and non-SEARCHers perceived acquiring and assessing evidence to be the most problematic steps of the EIDM process. For some, access to library research, research databases and availability of local data were challenging; some also found it difficult to understand the literature and to assess the different types of evidence (*e.g.*, scientific versus grey literature).

Our research led to two major findings:

There were marked changes in the attitudes of others in the organizations toward the use of EIDM, and these changes were reflected, in part, in the language used to discuss evidence and decision making. The following quotations support this finding:

C_F03: ‘There was a growing and emerging sensitivity to the need for research and the need to use evidence and in the competency and application of the tools to use evidence. So, I absolutely from the beginning saw a great growth.’

Mc_S04: ‘That kind of thing takes a long time to change, but you can see it’s spreading. You can hear it in the language as people talk about a new thing.’

Across all sites, change was very limited in terms of the skills acquired by others in the organizations for engaging in EIDM. The following quotation supports this finding:

H_F01: ‘It is almost like changing the way they work, which is difficult. So, I think it is a much more iterative long-term process to get you there.’

While the non-program interviewees at all six sites reported marked changes in organizational attitudes and the language, they found it more difficult to gauge changes involving the skills required to use evidence in decision making by other people. Except on rare occasions, such changes seem to have been confined to the level of attitude toward EIDM.

At EXTRA sites, the fellows seem to have influenced their colleagues during their program participation; this influence came about primarily through the intervention projects, which increased the fellows’ organizational visibility. SEARCHers also had an impact on the use of EIDM among their colleagues; however, the results differed somewhat from those at the EXTRA sites: while SEARCHers had a similar impact on their colleagues’ attitude and language changes, we did not observe any direct effect on the conversion of skills.

### Factors that facilitated or impeded a program’s organizational impact

We also sought to understand the factors that facilitated or impeded the transfer of EXTRA fellows’ or SEARCHers’ skills to other people in their organizations. As discussed earlier, Nonaka’s framework [8,10,16] provided us with a theoretical base for understanding this knowledge conversion process (see Table 4).

**Table 4:** Utility and contextual conditions of the four knowledge-conversion modes

**Conversion modes**	**Utility**	**Contextual conditions**
Socialization	• Gain local knowledge	• Trainee’s leadership skills
	• Strengthen attitudes	• Trainee’s role (mid-/senior-level manager)
	• Gain credibility	• Structure of the training program
		• Existence of collaborative network
Externalization	• Voice engagement with EIDM (conversion of attitudes)	• Trainee’s leadership
	• Show skills in the use of EIDM	• Scope and relevance of intervention project
		• Organizational communication culture
Combination	• Necessary for conversion of skills	• Collaborative networking
		• Learning culture and practices
		• Organizational leadership and support
		• Motivation to engage in team work
		• Flexible organizational arrangements (*i.e.*, decentralization of decision-making)
Internalization	• A first step toward routinization of the use of EIDM	• Learning processes and practices
		• Skills and knowledge resources in the organization
		• Organizational upheaval
		• CEO leadership

To begin with, our analysis also showed that certain external factors can affect the spread and use of EIDM within an organization. Interviewees identified the following external factors—that is to say, factors not directly related to the EXTRA and/or SEARCH Classic programs—as having influenced the use of EIDM in their organizations: the growing importance of quality improvement at the clinical level; the intensification of accountability pressures as individuals and organizations are increasingly required—by governments and public opinion—to justify their decisions; and the escalation of institution-level pressure as the use of EIDM becomes a norm for senior managers.

Some intervention projects seem to have had a more extensive impact on the organizational use of EIDM than others. In order to understand those influential factors, we focused on four ‘tracers’; these were particularly well-documented projects that had been described in detail by a training program participant as well as at least one colleague and one supervisor. Because we had more than one person speaking about the same project, we were able to examine events from different perspectives. Out of our research on these four tracers, we developed four narratives: three documented examples of successful organization-level EIDM knowledge conversion, and a fourth that was an example of a less successful process. All four narratives allowed us to learn from the participants’ experiences and to identify the factors that facilitate or impede knowledge conversion. The four modes of context-specific knowledge conversion discussed earlier—socialization, externalization, combination, internalization—shed further light on how dissemination occurred from participants to their colleagues.

### Socialization

As evidence of socialization, we looked to see whether EXTRA fellows and SEARCHers were involved in meaningful social interactions with others in their organizations (before, during and after their training). Those interactions, we theorized, would enable the fellows and SEARCHers to gain a better understanding of their organizations’ use of EIDM, to strengthen their attitudes toward the use of EIDM and to gain credibility with their colleagues.

We observed that all the fellows and SEARCHers participated in the socialization process, albeit with varying degrees of intensity. It is important to note that all these individuals worked in dynamic environments in which interactions and observations were numerous; indeed, interacting and sharing information with others were requirements of their management positions. The following quotations support our findings relevant to understanding socialization:

H_F06: ‘Face to face, asking people what their experiences were, how do they use evidence, you know, just enquiring whether or not people were aware of any existing frameworks.’

A_F02: ‘So, I actually did quite a bit of homework with the executive, in terms of meeting with them, [asking], ‘What are some of the key issues we’re facing? What research would you like to see? What might be of interest? What are we already started on and could we finish with?”

In addition, our analysis showed that the following conditions appear to affect socialization:

An individual’s leadership skills: These refer to an individual’s ability to motivate people to use EIDM. For example, one person talking about his EXTRA-fellow colleague said, ‘I'll give him credit, he will put pen to paper and get things out and publish something. … He brings that passion that just gets me fired up.’

An individual’s role (mid- or senior-level manager): Trainees’ managerial roles seemed to affect the intensity of the socialization process. Some trainees had central roles in their organization, while others were not perceived as change agents. For example, one trainee did not hold a senior administrative position; instead, he came from the medical sector, and we can assume he had fewer opportunities to socialize with other colleagues and administrators. He reported, ‘I thought it would be fairly easy, but it was actually quite difficult. It was seen as an imposition.’

Training program structure: Program structure includes delivery mode, instructional style and content. According to many trainees, program structure had an effect on the socialization process. More specifically, some perceived that management and leadership training helped them engage with other colleagues; for example, one person said, ‘I think the skills I learned were more the administrative people management. The change management stuff was just great! … That’s probably been my biggest benefit from the EXTRA program.’

Existence of a collaborative network: In some organizations, people had robust collaborative networks. The programs also enhanced collaboration between colleagues and helped foster new networks. One person noted, ‘We had [a] network … within our small group. … So that you do feel like you can ask someone, you don’t feel like you’re lost or you can’t go ahead. I think that’s part of that confidence.’

### Externalization

As evidence of externalization, we looked to see whether EXTRA fellows and SEARCHers found formal opportunities—vehicles that would enable fellows and SEARCHers to voice their engagement with EIDM and to demonstrate their skills—to communicate with their colleagues about their attitudes and skills. Those formal opportunities could include meetings, seminars, presentations and publications. Externalization would also, we believed, be useful for transferring attitudes; in that light, intervention projects would provide good externalization opportunities. All the EXTRA fellows and SEARCHers made presentations about their individual or group projects; however, we observed that the externalization process was stronger with EXTRA projects. The nature of a particular project (*e.g.*, literature review for SEARCH) was seen by several participants to contribute to the opportunities to externalize their skills and knowledge:

Mc_F01: ‘We presented it [*i.e.*, the project] in a number of forms. We had a number of major learning sessions or workshops. We also created … [and] we used a lot of vehicles for communicating the results back to people, because one of the things we really learned is you could be doing better but if the front line staff doesn’t actually know it, they feel like they’re wasting their time.‘

H_F02: ‘I did presentations, but I also sent out information in written form. I also had one-to-one, face-to-face conversations with key people that I thought could influence the change.’

DT_F03: ‘I actually had a steering committee for the dissemination … all of those players within those three organizations are also the main decision makers in this region, so they all had access to the information and, like I said, I did a digital story, so I quite often would go and show the digital story off the start of any presentation or dissemination that I did.’

Keeping in mind that the scope of an intervention project seemed to be a catalyst for externalization, we also discovered that the following conditions influenced externalization: an individual’s leadership abilities, a project’s scope and relevance to organizational’ priorities and an organization’s culture of communication.

### Combination

As evidence of combination, we looked to see whether EXTRA fellows and SEARCHers involved others in the actual practice of EIDM, most importantly in contexts not directly related to their projects. On the whole, we observed scant combination; that is, we noticed few interactive and constructive processes other than in the three successful projects that we studied in greater depth.

For example, one project on DVT resulted in the development of a new evidence-based protocol for that condition. In fact, it was more than just a protocol that was put in place, it was a new way to change practice based on evidence. The whole change process involved a lot of discussion and exchange among different parties. The trainee’s senior-level management team and CEO were strongly committed to his project, and he had organizational support to make decisions in order to change DVT practice. Another important factor in his success was the trainee’s adoption of a process of collaborative and collective teamwork that helped involve other people and to transfer skills and knowledge. Because of his leadership and knowledge of the specific context, he was able to motivate people to get involved in the project. There seems to have been a lot of respect and listening in his approach, and he also built in time for reflection and ongoing improvement of the protocol. This helped other people relate to the new protocol and adopt it. The new protocol also involved changes in other professionals’ responsibilities, a transformation that was supported by the CEO and the organization’s senior leaders, and that showed that the organization had enough flexibility in its structures to allow changes. In the trainee’s words:

‘We gave the information and then we put it up on the wall. And then people would come in and write on it and then we’d go every few days to the different people and say, ‘What’s come up?’ And so we did rounds with radiology twice, [and] we went to the [academic] rounds where they all discussed it.

‘Implementing a transition tool, which is a communication tool, when patients are moved from the emergency department to the ICU and to other units: this came out of a master’s student’s research project … and so they presented their results and their analysis and, based on the research that they had done, then the clinicians are taking that and saying, ‘OK, so now we’re going to put this in place, we’re going to pilot it and we’re going to evaluate the impact of it.”

Based on our findings, we conclude that the following conditions influence the success of combination: collaborative networking: the extent of a trainee’s collaboration with people inside and outside his/her organization; learning culture and practices: the presence of an organizational learning culture involving a cycle of action and reflection; organizational leadership and support for EIDM and practice changes: motivation for team work: the perception that team work is beneficial; and flexible organizational arrangements (organicity): decentralization of the decision-making process, which ensures more people are able to use EIDM.

### Internalization

As evidence of internalization, we looked for changes in the practice of EIDM in organizations, whereby individuals other than the EXTRA fellows and SEARCHers employed EIDM. While we found changes in attitudes toward EIDM in the organizations we studied, it was much harder to detect evidence of skill conversion. Based on our findings, we conclude that the following conditions affect internalization: learning processes and practices; an organization’s skills and knowledge resources; and CEO leadership in promoting EIDM.

## Discussion

### Principal findings

In our research, we sought first to determine the nature and extent of the impact on an organization of having a number of mid- and senior-level managers trained through the EXTRA and SEARCH Classic programs. We hypothesized that individual learning could spread within an organization through the interaction of tacit and explicit knowledge via four modes of knowledge conversion. We found that the impact could primarily be felt in close circles; that is, in trainees’ immediate work environments. Our results showed a change in the language used by colleagues and a new awareness and sensitivity about the use of evidence in decision making. The conversion of attitudes was found to be easier to achieve than the conversion of skills.

Our project also analyzed—again through the four modes of knowledge conversion—the organizational processes by which a training program’s impact occurs. Our results show that, although socialization and externalization were common in all cases, a lack of combination impeded the conversion of skills. However, some degree of combination did occur in cases where the trainees were able actively to involve others in the process of using EIDM. This finding is compatible with Nonaka *et al.*’s [32] view of combination as usually the most problematic mode of knowledge conversion because of difficulties associated with involving other organizational members.

We also identified several individual, organizational and program design factors that facilitated and/or impeded the dissemination of the attitudes and skills gained by trainees to other organizational members (Table 5). Among those factors, the following had the most influence:

1. The individual characteristics of EXTRA fellows and SEARCHers (*e.g.*, skills, leadership, centrality in their organizations, personal networks) affected their capacity to drive the four modes of knowledge conversion and to transfer attitudes and skills to others.

2. The organizations’ skills and knowledge stocks influenced people’s ability to engage in learning new information and approaches. A ‘knowledge stock’ is the level of accumulated knowledge in an organization [18,8].

3. CEOs’ leadership facilitated learning processes through various strategies aimed at enabling EIDM.

4. A strong communication and learning culture, collaborative intra-organizational networking, and flexible organizational arrangements appeared to be requirements for engaging in the four modes of knowledge conversion necessary for translating individual knowledge into organizational knowledge.

5. Certain program design characteristics influenced knowledge transfer; for example, the strategic relevance of trainees’ projects to their organizations, the extent of support provided by an organization throughout individuals’ training periods, the degree to which a program focused on cultivating leadership in EIDM, and the creation of communities of practice among trainees.

**Table 5:** Factors that influenced the use of EIDM and knowledge conversion

**Individual characteristics**	**Organizational contexts**	**Program characteristics**
Skills and knowledge in EIDM	Skills and knowledge stock	Scope and relevance of intervention project (team- based projects)
Strength of leadership	CEO leadership	Mentoring and support during and after
Central role in the organization	Communication culture	Program focus
Personal network	Learning processes and practices	Organizational involvement in the program
	Collaborative networking	Intensity and scope of the ongoing community of practice
	Organizational commitment to and strategies that support EIDM	Targeted clientele
	Organizational condition (*e.g.*, stable or in a state of change)	
	Flexible organizational arrangements	

Healthcare organizations and health system leaders have made significant efforts to encourage the use of evidence in decision making, believing it will lead to more effective organizational management; as a result, many different strategies have been formulated to facilitate healthcare managers’ use of EIDM. Because the impact of those efforts on actual practices within organizations has been unclear, our theory-driven evaluation showed that enhancing knowledge capacity in an organization through an educational intervention is a major challenge, and that it cannot be accomplished through a single strategy.

As previous studies have discovered [31,33], factors before, during and after training can influence the extent of skills and knowledge transfer. Our evaluation went further than previous research by revealing the influence—both positive and negative—of specific organizational factors on extending the impact of training programs.

### Practice implications

Our results show that *combination* is the sore spot in the conversion of individual skills. It is essential, therefore, that individual training programs design ways to help participants engage others in their organizations. This could be addressed directly in curricula and/or it could take the form of other strategies to be designed and unfolded in partnership with trainees’ organizations. The role of mentoring by organizational members throughout training could also be strengthened in order to increase the direct involvement of additional organizational participants in EIDM processes.

Managers in complex organizations must deal with structural changes taking place within turbulent environments. In the course of our study, this proved to be a constant reality. It affected the EXTRA fellows’ and SEARCHers’ use of EIDM, as well as their capacity to influence others, and it was a particularly severe challenge at the SEARCH sites. Among EXTRA fellows, turbulent environments influenced the conduct of some of their intervention projects. Environmental turbulence does not necessarily lead to failure in the conversion of skills and attitudes; however, training programs’ curricula should address how to adapt and make changes in chaotic circumstances. Organizations should also address their ability to learn and adapt rapidly to changing circumstances.

We believe the results of our theory-driven evaluation will be of interest for decision makers and program developers alike. Our findings suggest that expecting change to occur as a result of training programs is unrealistic unless an organization is aware of and develops strategies to deal with the multiple complexities involved in converting individual-level skills and knowledge to skills and knowledge that are held at the group and organizational levels.

## Conclusions

EXTRA and SEARCH Classic were based on the assumption that if enough people were trained in EIDM an organization would get to a tipping point, at which stage EIDM would disseminate and become organizational. This assumption was founded on the concept of critical mass being a fundamental factor required to initiate and sustain changes in an organization’s application of EIDM (we did not, however, measure ‘critical mass,’ as our intent was to understand how knowledge gained through individual programs could spread beyond the trainees and spread within the organization). Our results show that the number of people trained is not a sufficient condition to assure organizational dissemination of knowledge. We also revealed several limitations of the EXTRA and SEARCH Classic program assumptions. To begin with, the transfer of attitudes was achieved when EXTRA fellows and SEARCHers held central and/or leadership positions in their organizations. In addition, on the rare occasions it occurred, skills transfer was limited to the close circle around a fellow or SEARCHer. Extrapolating from this finding, we conclude that a high number of program participants may be required to achieve an organization-wide transfer of EIDM skills, but will in and of itself not be a sufficient condition for success. Involvement of other people in an organization, as well as high quality, active communication, seems to be essential for organizational changes to occur.

Our research did not show a distinction in knowledge conversion between mid- and senior-level managers. The main determinant affecting knowledge conversion seemed to be the centrality of the trainees’ positions within their organizations. For knowledge conversion to occur, a trainee had to be in a role that involved a high degree of social interactions, to be directly involved in decision making and to be exercising leadership.

We also uncovered the critical role played by organizational resources. According to our findings, rural organizations benefited the most from having managers enrolled in training programs. That was because those programs provided continuing education opportunities in regions that had no other such opportunities (which is not the case in larger urban centers). As well the programs drove the development of research projects specific to rural regions.

The EXTRA program enrolled both individuals and teams. In this respect, our comparative analysis showed that simultaneously training multiple individuals from the same organization and having them work together on a project focused on achieving a common goal seems to provide more opportunities to socialize, externalize and combine knowledge.

### Availability of supporting data

The data sets supporting the results of this article are available in the following report: Champagne F, Lemieux-Charles L, MacKean G, Reay T, Suarez-Herrea JC, Dubois N, Duranceau MF: *Knowledge Creation in Healthcare Organizations as a Result of Individuals’ Participation in the EXTRA and SEARCH Programs*. Ottawa: Canadian Foundation for Healthcare Improvement; 2011.

## Abbreviations

CEO: Chief executive officer; CFHI: Canadian foundation for healthcare improvement; CHSRF: Canadian health services research foundation; EIDM: Evidence-informed decision making; EXTRA: Executive training for research application; SEARCH: Swift, efficient application of research in community health.

## Competing interests

The authors declare that they have no competing interests.

## Authors’ contributions

FC, LLC, GMK, and TR were responsible for the conception of the study and, together with MFD, planned the process analysis. FC, LLC, and MFD formulated and composed the questionnaires. MFD performed the daily work associated with study inclusion and data collection. All authors were involved in writing the manuscript and all authors read and approved the final version.
